# Local Immune Response in *Helicobacter pylori* Infection

**DOI:** 10.1097/MD.0000000000003713

**Published:** 2016-05-20

**Authors:** Derya Kivrak Salim, Mehmet Sahin, Sadi Köksoy, Haydar Adanir, Inci Süleymanlar

**Affiliations:** From the Department of Medical Oncology (DKS), Faculty of Medicine, Akdeniz University, Antalya; Faculty of Health Sciences (MS), Kahramanmaras Sutcu Imam University, Kahramanmaras; Department of Microbiology (SK); and Department of Gastroenterology (HA, IS), Faculty of Medicine, Akdeniz University, Antalya, Turkey.

## Abstract

There have been few studies concerning the cytokine profiles in gastric mucosa of *Helicobacter pylori*–infected patients with normal mucosa, chronic gastritis, and gastric carcinoma (GAC).

In the present study, we aimed to elucidate the genomic expression levels and immune pathological roles of cytokines—interferon (IFN)-γ, tumor necrosis factor (TNF)-α, interleukin (IL)-4, IL-6, IL-10, transforming growth factor (TGF)-β, IL-17A, IL-32—in *H pylori*–infected patients with normal gastric mucosa (NGM; control), chronic active gastritis (CAG), and GAC. Genomic expression levels of these cytokines were assayed by real-time PCR analysis in gastric biopsy specimens obtained from 93 patients.

We found that the genomic expression levels of IFN-γ, TNF-α, IL-6, IL-10, IL-17A mRNA were increased in the CAG group and those of TNF-α, IL-6, IL-10, IL-17A, TGF-β mRNA were increased in the GAC group with reference to *H pylori*–infected NGM group.

This study is on the interest of cytokine profiles in gastric mucosa among individuals with normal, gastritis, or GAC. Our findings suggest that the immune response of gastric mucosa to infection of *H pylori* differs from patient to patient. For individual therapy, levels of genomic expression of IL-6 or other cytokines may be tracked in patients.

## INTRODUCTION

Gastric carcinoma (GAC) is the fifth most common cancer in the world with 9% of total cancer mortality. Most of the noncardia GACs are caused by *H pylori* infection.^1,2^ After *H pylori* colonization in the stomach, which is often asymptomatic, polymorphonuclear leukocytes, macrophages, and lymphocytes infiltrate the mucosa. This inflammation together with some bacterial toxins and constituents could cause gastroduodenal ulceration, GAC, and mucosa-associated lymphoid tissue lymphoma. The molecular mechanisms of local immune response initiated by *H pylori* are complex, but it is believed that cytokines produced by both immune and non-immune cells amplify the ongoing inflammation.^3^ To date, the pathogenesis of *H pylori*-related chronic gastritis and the mechanism responsible for the change into neoplasm are still debated. The carcinogenicity of *H pylori* through the local and systemic immune response and the roles of cytokines and chemokines have been investigated in many researches. If we could define such cytokines and chemokines, which are related to the increased GAC risk in the premalignant period, we may explain the chronic inflammation-mediated neoplastic changes and use them as early predictive diagnostic molecular markers.

There are 2 groups of immune response, Th1 and Th2, and they can be distinguished from one another by cytokine profiles. Th1 proinflammatory cytokines are interleukin (IL)-2, IL-12, interferon (IFN)-γ, and tumor necrosis factor (TNF)-α; Th2 anti-inflammatory cytokines are IL-4, IL-6, IL-10, and transforming growth factor (TGF)-β.^4^ Th1 immunity is important in antitumor activity and Th2 immunity is dominant in advanced carcinomas because the Th1/Th2 ratio is important for an antitumor effect. Th1 immunity is more favorable in anti-tumor immunity than Th2 immunity.^5,6^ In this study, we investigated the cytokines representing Th1 and Th2 immunity, which interact with each other. We looked for new cytokine expression profiles in gastric mucosal inflammation-mediated carcinoma.

Recently, new molecular markers have led to new novel diagnostic strategies. After the discovery of tumor suppressor gene inactivation, changes in angiogenic factors, pathways, and gene expression and transcription factors in signaling, monoclonal antibody-based (targeted) therapies, cytokine therapies, adoptive immunotherapy, etc have been developed.

Quantitative cytokine differences in *H pylori*–induced gastric mucosal inflammation may play a pivotal role in determining the various clinical outcomes of this infection. This article focuses on the interactions between cytokine mediators and clinical outcomes (gastritis, malignancy etc.) in *H pylori* infection.

## MATERIAL AND METHODS

### Subjects and Tissue Samples

To evaluate the utility of IFN-γ, TNF-α, IL-4, IL-6, IL-10, TGF-β, IL-17A, and IL-32 cytokine mediators as markers associated with increased GAC risk, we investigated the gastric tissues of 93 dyspeptic patients infected with *H pylori* in whom a diagnosis had been made using a rapid urease test. The data and specimens were collected between June 2011 and February 2012 in Akdeniz University School of Medicine Hospital.

Exclusion criteria were as follows: the use of nonsteroidal anti-inflammatory drugs, proton pump inhibitors (PPIs), histamine H_2_-receptor antagonists, antibiotics, immunomodulatory drugs, steroids, antacids, chemotherapeutic drugs, *H pylori* eradication treatment within 4 weeks before the present study, severe concomitant disease, previous peptic ulcer disease, gastric cancer or other neoplasms, and comorbid conditions that might interfere with immunity or immune response, including diabetes mellitus, chronic renal failure, chronic liver failure, autoimmune diseases, chronic alcohol intake, and allergy.

The gastric tissue samples were collected from the prepyloric region after 8 hours of fasting. During endoscopy, at least 3 biopsy specimens were taken for each patient in each group. These included 1 group for the CLO test, 1 fixed in formol for histopathological examination, and 1 for real-time PCR (RT-PCR) analysis. CLO test–positive patients were included for further histopathologic and genomic expression evaluation. The study groups were determined by histopathology. Any histological abnormality was accepted as gastritis and marked lymphoplasmacytic inflammation with the presence of neutrophils was regarded as chronic active gastritis (CAG). GAC is classified according to World Health Organization histological classification. The gastric tissue samples for RT-PCR were collected in 2-mL tubes and kept in RNAlater solution at −20°C until the time of assay. Tissue samples were thawed by using magNA Lyser Green Beads (Cat. No: 03 358 941 001) and magNA Lyser Instrument (Cat. No: 03 358 976 001) of Roche Trademark. After homogenization, the RNA material from gastric tissues was isolated according to the protocol of PureLink^TM^ RNA Mini Kit (Cat.no.1218018A). The RNA content was measured by the PerkinElmer Lambda Bioplus spectrophometer at 260 nm UV absorbance. The cDNA material was produced from RNA material using commercially available Applied Biosystem High Capacity RNA-to-cDNA Kit (Product P/N 4387406). The cytokine gene expression levels were measured using commercially available gene expression assay kits. Here, 10 μL of TaqMan Universal PCR master mix kit, 8 μL of nuclease-free H_2_O, 1 μL of gene expression kit (involved cytokine), and 1 μL of cDNA were mixed and put into a plate for Roche LightCycler 480 PCR equipment; then, RT-PCR was performed. PCR amplification was done for cytokines, IFN-γ, TNF-α, IL-4, IL-6, IL-10, TGF-β1, IL-17A, IL-32, and beta-actin as a reference gene. The cytokine gene expression levels were measured with an advanced relative quantification method in LightCycler 480 PCR software programme. Values were normalized and expressed as relative to normal gastric mucosa (NGM) group. According to histopathological groups (NGM, CAG, and GAC) IFN-γ, TNF-α, IL-4, IL-6, IL-10, TGF-β1, IL-17A, and IL-32 cytokine gene expression levels were calculated by using the RT-PCR method prospectively. The study protocol was approved by the local research ethics committee.

### Statistical Analysis

Statistical analysis was carried out using the “Statistical Package for the Social Sciences” (SPSS) Statistics version 20.0 for Mac OSX (NY, USA). Also, the GraphPad Prism Software (San Diego, CA) program was used for the comparison of cytokine mRNA levels. Real-time PCR data, which were not normally distributed, were analyzed with Kruskal-Wallis for comparison between 3 groups. Descriptive statistics, including mean and SEM, were used to describe the sample. Values of 3 groups were expressed as mean ± SEM and given as figures. *P* < 0.05 was considered to indicate a statistically significant difference.

For categorical data, Pearson *χ*^2^ test and Fisher exact *χ*^2^ test were used. A *P* value of <0.05 was accepted as statistically significant. The variables, which were determined significant in univariate analysis (Kruskal-Wallis, *P* < 0.05 as parameters), were evaluated by logistic regression analysis to understand which is an independent predictor of GAC risk alone. For this analysis, firstly continuous variables (quantity that has a changing value) were dichotomized on the basis of median values (age ≤55 or>55 form and IL-6 ≤22 or >22) because there was no priori rationale. These dichotomized parameters were used in multivariable regression analysis. Multinomial regression analysis was performed with the forward entry method because there is not a similar analysis carried out previously and there is no knowledge about which parameters will be independent variable. Results were reported as odds ratios (ORs) and 95% confidence intervals (CIs).

## RESULTS

This study concerned 43 females (46.2%) and 50 males (53.8%) with the age range 21 to 70 years (median age: 56, mean age: 52.77 ± 13.78). Among the 93 patients infected with *H pylori*, 32 (34.4%) had NGM, 33 (35.5%) had CAG, and 28 (30.1%) had GAC. Table 1 shows the demographic data of the study groups.

**TABLE 1:**
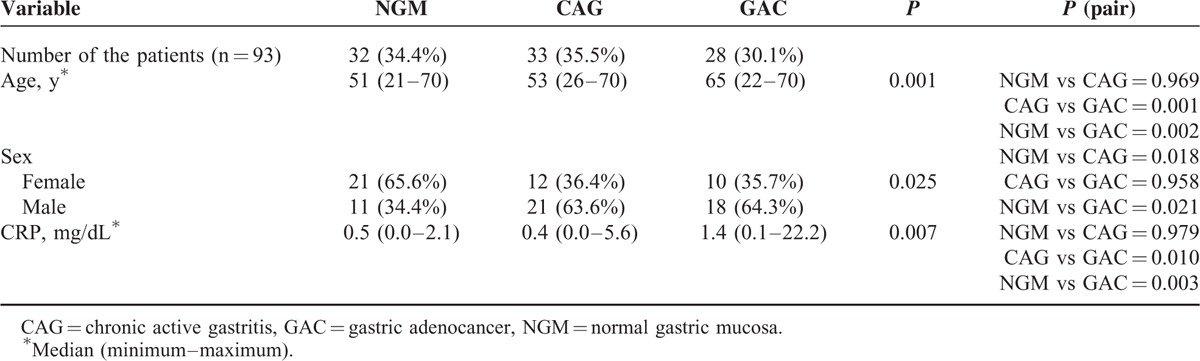
Demographic Data of the Study Groups

There was a significant difference between the groups (NGM, CAG, and GAC) with regard to age (*P* = 0.001) (Table 1). The median age in the GAC group was statistically higher than in the NGM and CAG groups. A statistically significant difference between the NGM and CAG/GAC cases in terms of sex is also found (*P* = 0.025) (Table 1).

### Relative mRNA Levels of Cytokines

**FIGURE 1:**
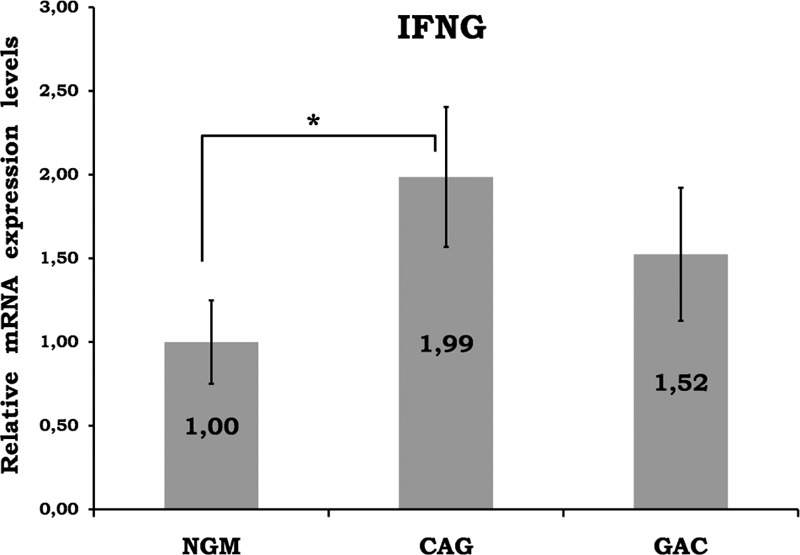
Relative gene expression levels of IFN-gamma. The results were expressed as mean ± SEM. ^∗^*P* < 0.05. CAG = chronic active gastritis, GAC = gastric adenocancer, IFN-γ = interferon-gamma, NGM = normal gastric mucosa.

**FIGURE 2:**
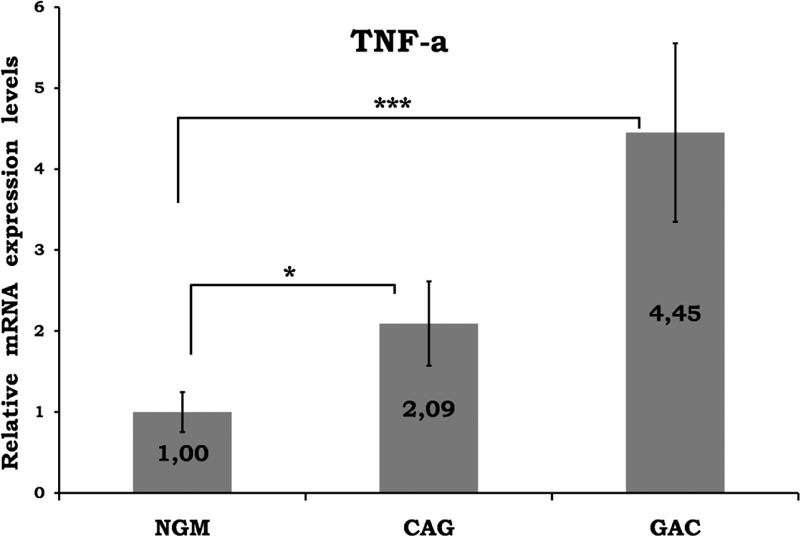
Relative gene expression levels of TNF-α. The results were expressed as mean ± SEM. ^∗^*P* < 0.05 and ^∗∗^*P* < 0.001, respectively. CAG = chronic active gastritis, GAC = gastric adenocancer, NGM = normal gastric mucosa, TNF-α = tumor necrosis factor alpha.

**FIGURE 3:**
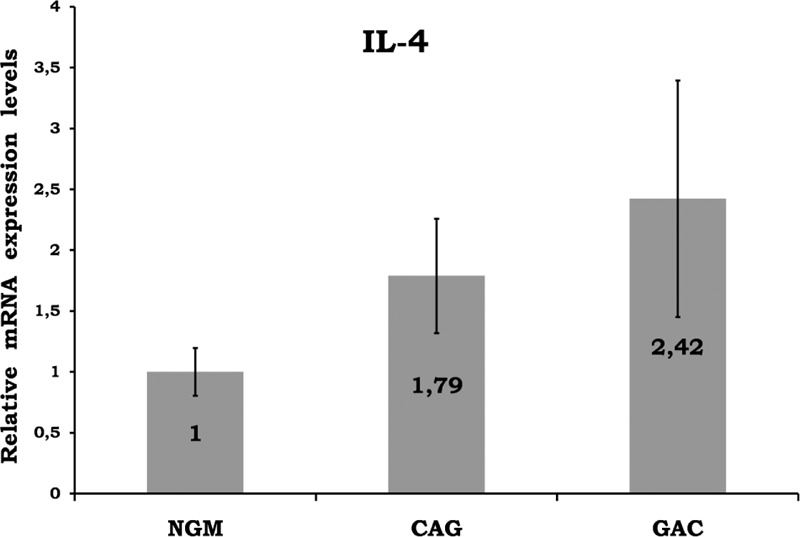
Relative gene expression levels of IL-4. Any significant result was not observed among the groups. CAG = chronic active gastritis, GAC = gastric adenocancer, IL-4 = interleukin-4, NGM = normal gastric mucosa.

**FIGURE 4:**
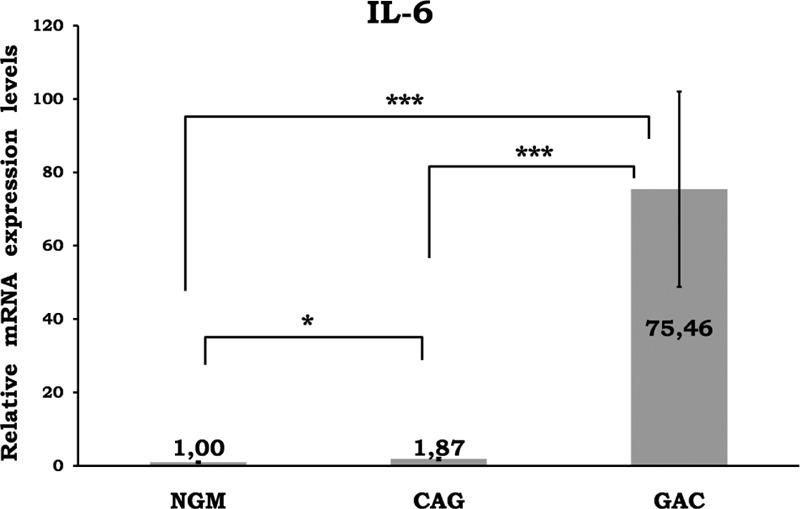
Relative gene expression levels of IL-6. The results were expressed as mean ± SEM. ^∗^*P* < 0.05 and ^∗∗^*P* < 0.001, respectively. CAG = chronic active gastritis, GAC = gastric adenocancer, IL-6 = interleukin-6, NGM = normal gastric mucosa.

**FIGURE 5:**
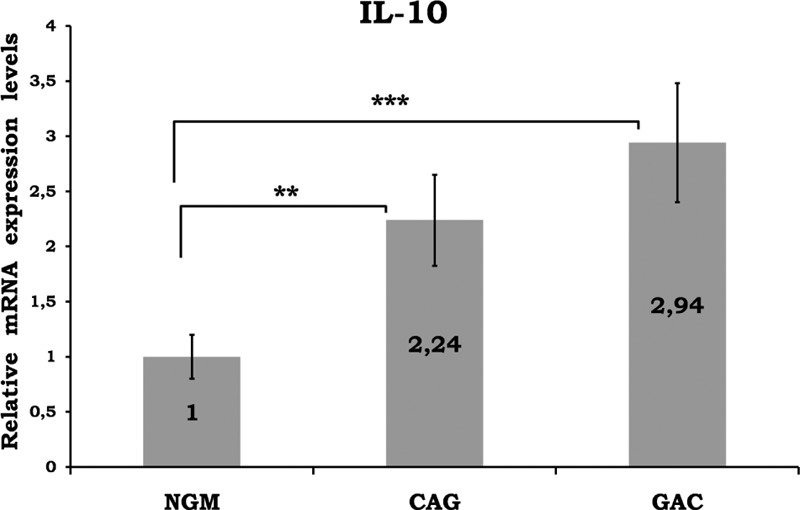
Relative gene expression levels of IL-10. The results were expressed as mean ± SEM. ^∗^*P* < 0.01 and *P* < 0.001, respectively. CAG = chronic active gastritis, GAC = gastric adenocancer, IFN-γ = interferon-gamma, NGM = normal gastric mucosa.

**FIGURE 6:**
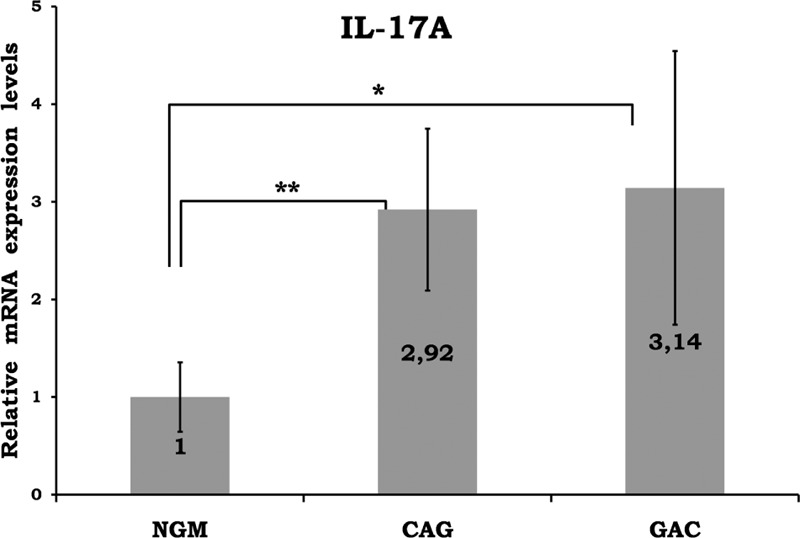
Relative mRNA levels of IL-17A. The results were expressed as mean ± SEM. ^∗^*P* < 0.05 and ^∗∗^*P* < 0.01, respectively. CAG = chronic active gastritis, GAC = gastric adenocancer, IL-17A = interleukin-17A, NGM = normal gastric mucosa.

**FIGURE 7:**
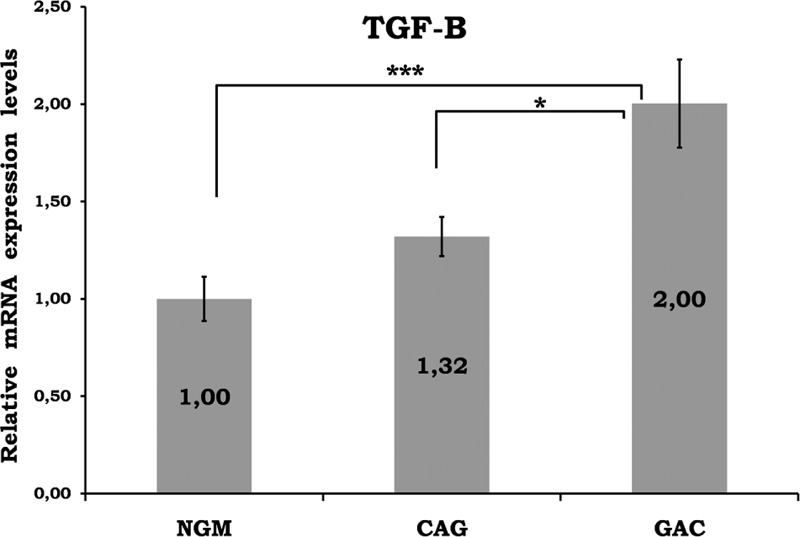
Relative gene expression levels of TGF-β. The results were expressed as mean ± SEM. ^∗^*P* < 0.05 and ^∗∗^*P* < 0.001, respectively. CAG = chronic active gastritis, GAC = gastric adenocancer, NGM = normal gastric mucosa, TGF-β = transforming growth factor-gamma.

**FIGURE 8:**
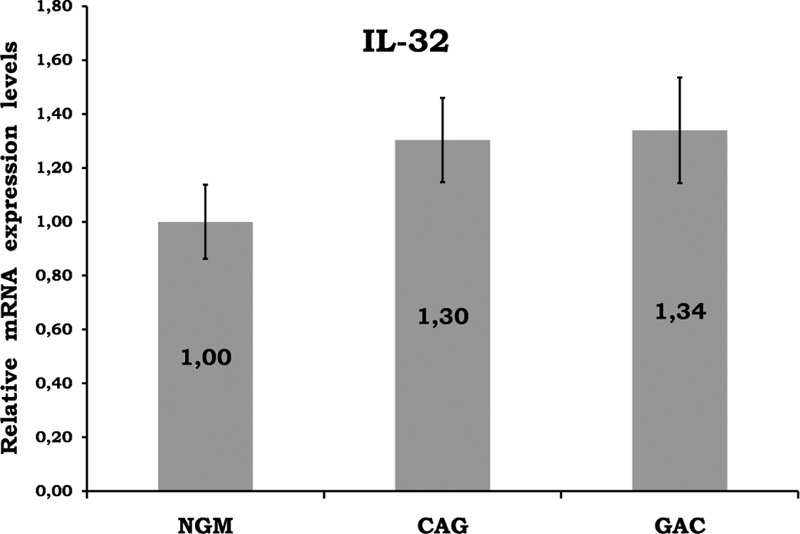
Relative gene expression levels of IL-32. No significant result among the histopathological groups. CAG = chronic active gastritis, GAC = gastric adenocancer, IL-32 = interleukin-32, NGM = normal gastric mucosa.

Mean data of CAG and GAC shown in Figures 1–8 are values that are relative to mean of NGM group that is reduced to 1. Figures 1–8 show the differences of the mucosal genomic expressions of cytokines in histopathological groups. However, statistically used data were “RT-PCR results” calculated by LC-480 software. Data were expressed as mean ± SEM for all genomic expression levels.

Gastric mucosal IFN-γ, TNF-α, IL-6, IL-10, IL-17A mRNA genomic expressions were increased in *H pylori*–infected CAG compared with NGM groups (Figures 1, 2 and 4–6, respectively). The relative mRNA expressions of TNF-α, IL-6, IL-10, IL-17A, TGF-β were found to have a significant increase between GAC and control patients (NGM) (Figures 2 and 4–7). Although IL-4 and IL-32 increased in CAG and GAC histopathological tissues compared with NGM groups, any significant results were not evaluated (Figures 3 and 8). Median, minimum, and maximum values of cytokines’ genomic expression levels of 3 groups were presented in Table 2.

**TABLE 2:**
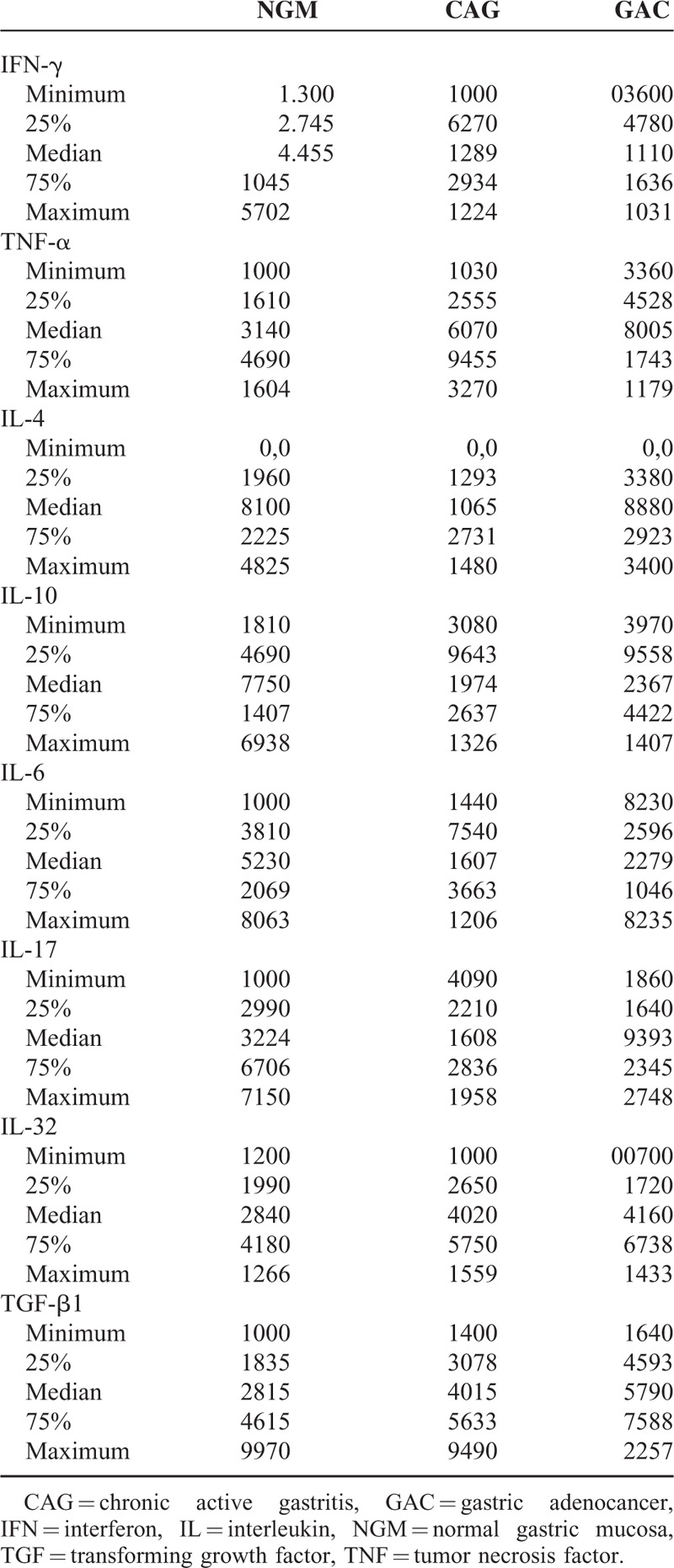
Relative Gene Expression Levels of Cytokines in NGM (Control), CAG, and GAC

In multinominal regression analysis, dependent variable was the histopathological groups. IFN-γ, TNF-α, IL-6, IL-10, TGF-β, IL-17A, age, and sex were included into the model for determination of independent predictors of the dependent variable. As a result, IL-6 >22 was found to be an independent factor. The relative genomic expression rate of IL-6 >22 was found to be higher in GAC development with reference to the control group (OR = 1509; 95% CI = 417–5456; *P* < 0.001).

In multivariate analysis of IL-6 expressions according to TNM staging (stage 0–stage 4) and grade of tumor in the GAC group, the median test was evaluated. There was no significant difference because there were no patients in stages 0 and 1; all patients were in advanced stages. In the advanced stages, IL-6 expression boosts with the stage 2, 3, 4 (*P* = 0.0081) (Table 3).

**TABLE 3:**
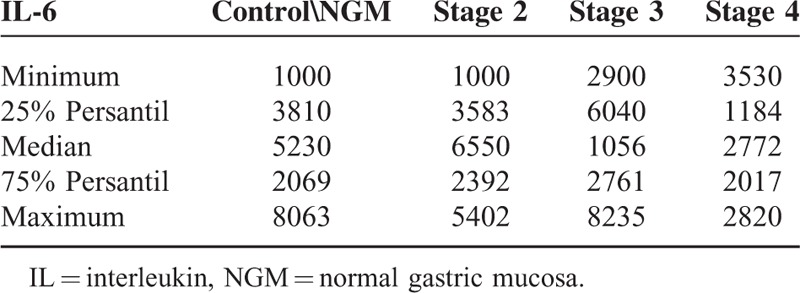
IL-6 expression in advanced TNM stages

As a result, when whole data were analyzed, the cytokine genomic expression levels, especially IL-6, were found to be increased in *H pylori*–infected CAG and GAC groups compared with *H pylori*–infected NGM.

## DISCUSSION AND CONCLUSION

Although most *H pylori*–infected people are asymptomatic, this infection may cause gastritis, duodenal ulcer, and GAC.^7,8^ In this study, we investigated the cytokines, which play major roles in Th1 and Th2 immunity. Our aim was to look for new cytokine genomic expressions in gastric mucosal inflammation-mediated neoplasm.

There have been few studies about the cytokine genomic expression profile in chronic gastritis. In one of these studies, cytokine gene expressions were investigated by RT-PCR in gastric biopsy specimens with chronic gastritis, and it was reported that although the histological severity of the gastritis was closely associated with *H pylori* infection, the positivity of IFN-γ and TNF-α cytokine mRNAs did not show a relationship with either *H pylori* infection or histological inflammation. They suggested that the gastric mucosa does respond in the same manner to all exogenous antigens immunologically including *H pylori*; IFN-γ and TNF-α do not play a role in the start of inflammation associated with this infection.^9^ IFN-γ mediates some responses to bacterial infection and autoimmune disease, and is also an important tumor suppressor. It is upregulated in the gastric mucosa by chronic *H pylori* infection; however, whether it plays a positive or negative role in inflammation-associated gastric carcinogenesis is unexplored. Tu et al^10^ mentioned antibacterial and antitumoral effect of IFN-γ by inhibiting gastric progenitor cells and inducing an autophagic programme.^10^ Additionally, Lindgren et al^2^ showed the decreased production of IFN-γ in the peripheral blood and gastric mucosa of GAC patients after stimulation with *H pylori* lysate. Hosseini et al investigated IFN-γ from 3 groups: GAC, nonulcer dyspepsia, and peptic ulcer patients. IFN-gamma gene expression was found to be similar in non-GAC dyspeptic patients, but it was significantly higher in GAC patients than in others.^11^ Our study showed significant elevated local mucosal genomic expression of IFN-γ in *H pylori*–mediated chronic active gastritis (*P* < 0.05). On the contrary, there was an insignificant decrease in IFN-γ genomic expression in the GAC group, which may also cause a decrease in tumor suppression effects (Figure 1).

In most of the recent studies, increased TNF-α levels have been shown in *H pylori*–infected gastric mucosa, which stimulate gastric dysplasia and GAC development.^12^ The role and quantity of TNF-α were frequently investigated in *H pylori* infection, but little is known about *H pylori*–infected GACs. In some studies, no relationship between *H pylori* and TNF-α was observed.^9^ Fan et al^13^ showed that higher levels of TNF-α production by antral mucosa cells in *H pylori* infection may reflect the mucosal infiltration by T lymphocytes and macrophages. In another study, it was reported that TNF-α may not participate in the development of inflammatory response by *H pylori*–induced gastric inflammation.^14^ However, with the eradication of *H pylori*, a decrease in chronic inflammatory infiltrate and TNF-α gene expression was shown.^15^ In addition, patients with *H pylori* (+) duodenal ulcers were observed to have a higher level of gastric mucosal TNF-α compared with *H pylori* (−) cases.^16^ Wu et al reported that TNF-α values, which were not independent prognostic indicators, were increased in the carcinoma group in contrast with benign gastric lesions and normal controls.^17^ Our results showed a significant difference in local mucosal genomic expressions of TNF-α between control (NGM) and CAG/GAC (*P* < 0.05 and *P* < 0.001, respectively) (Figure 2). There was an approximately 4.5-fold increase in the average of the GAC group. Medians of CAG and GAC were too close to be independent predictive markers of GAC.

IL-4, which is a Th2 cytokine, has not been related to either *H pylori* infection or histological inflammation.^9^ Hosseini et al investigated IL-4 in GAC, nonulcer dyspepsia, and peptic ulcer patients. Its expression was shown to have no significant difference between nonulcer dyspepsia and GAC patients, whereas it was significantly higher in peptic ulcer patients.^11^ In another study, IL-4 expressions were reported to be unrelated to both *H pylori* (+) and (−) groups.^18^ In the present study, we observed that IL-4 genomic expression was similar in NGM dyspeptic patients and GAC patients, whereas in CAG patients, it was insignificantly higher than others (Figure 3). Therefore, we propose that IL-4 does not play an etiological role in *H pylori* inflammation-related lesions and GAC.

Interleukin-6 (IL-6) is significantly associated with *H pylori* infection.^19^ It is both a proinflammatory and anti-inflammatory cytokine. It shows anti-inflammatory effects via the inhibition of TNF-α and IL-1. In one study, mucosal IL-6 levels were found to be significantly associated with the grade of inflammatory cell infiltration and the risk of *H pylori*-induced gastrointestinal disease development.^20^ Kabir et al^21^ mentioned that IL-6 expression was significantly increased (approximately 6-fold) in GAC patients. However, some studies showed no relation between *H pylori* infection and IL-6 mRNA expressions.^9^ IL-6 levels were found to be significantly higher in patients with GAC than in patients with benign gastric lesions and in normal subjects. Serum IL-6 levels correlated moderately with serum C-reactive protein levels and survival, but not as an independent prognostic indicator.^22^ In some GACs, IL-1 induces IL-6 as an autocrine growth factor.^23^ De Vita et al^24^ showed that IL-6 serum levels were elevated in advanced gastrointestinal carcinoma patients and correlated with both overall survival and time to disease progression. However, this was not an independent prognostic factor.

Our data support previous reports confirming that IL-6 levels are elevated in *H pylori* infection and GAC. We showed a significant difference in local mucosal genomic expressions of IL-6 among all 3 groups (Figure 4). There was an approximately 75-fold and 40-fold significant increase in the average of the GAC group compared with the NGM and CAG groups, respectively (*P* < 0.001) (Figure 4). In addition, IL-6 may also be involved in malignant transformation and tumor progression. It may start to rise in the premalignant period; however, future studies are needed.

IL-10 is an anti-inflammatory cytokine acting by way of the inhibition of IFN-γ, IL-1, IL-6, IL-8, and TNF-α. Increased IL-10 levels were reported in *H pylori*–infected gastric mucosa.^25,26^ There are also some studies insisting on no significant differences in IL-10 levels among *H pylori* infection.^27,28^ With regard to our results, we found significant differences in local mucosal genomic expressions of IL-10 between control and CAG/GAC (*P* < 0.01 and *P* < 0.001, respectively) (Figure 5). There was a 3-fold increase in the average of the GAC group. Increased IL-10 as an anti-inflammatory cytokine may suppress the cytotoxic antitumor T-cell response and may cause malignant transformation.

*H pylori*-induced mucosal inflammation results in high production of IL-17A, which causes neutrophil proliferation and accumulation in gastric mucosa.^29^ CD8^+^ T cells producing IL-17A (Tc17 cells) have been identified in tumors. In one study, percentages of Tc17 cells in GACs were found to be associated with survival times of patients. These cells promote chemotaxis of myeloid-derived suppressor cells, which might promote tumor progression.^30^ Kennedy et al^31^ suggested that the increased expression of Th17 is not related to the molecular pathogenesis of gastric carcinogenesis. Caruso et al^3^ found a decrease in IL-17A levels in *H pylori*-infected gastric biopsies. In another study, it was suggested that IL-17A expression was significantly lower in GAC patients as compared with healthy individuals.^32^ Chen et al reported that GAC patients expressing high levels of IL-17A intratumorally had significantly better 5-year overall survival probability than those expressing lower levels of IL-17A. They also revealed that intratumoral IL-17A expression was an independent factor for 5-year overall survival.^33^ It was demonstrated that long-lasting and unrelieved Th17 inflammation in *H pylori* infection may represent an immunopathological condition that links the infection and GAC.^34^

Our results showed a significant difference in the mucosal genomic expression of IL-17A between the NGM and CAG/GAC groups (*P* < 0.01 and *P* < 0.05, respectively) (Figure 6). There was an approximately 3-fold increase in the average of the GAC group and a 2.9-fold increase in the average of the CAG group. In this regard, recent studies, including ours, showed that IL-17A was overexpressed in the gastric mucosa of *H1 pylori*-infected patients and its levels were significantly elevated in parallel with the severity of lesions (benign lesions to malign ones).

TGF-β is an angiogenic factor, which is an essential mediator for preventing abnormal mucosal proliferation or for suppressing carcinogenesis in the gastrointestinal tract.^35^ TGF-β is increased in gastritis, peptic ulcer disease, and GACs.^36^*H pylori* infection is associated with depressed gastric mucosal TGF-β.^37^ Similar to previous studies, we determined a significant difference in the genomic expression of TGF-β between NGM and GAC (*P* < 0.001), and between CAG and GAC groups (*P* < 0.05) (Figure 7). There was a 2-fold increase in the average of the GAC group. These findings are in line with the suggestion that gastric mucosal TGF-β levels can be used to determine the outcome after *H pylori* infection.

Interleukin-32 (IL-32) is a proinflammatory cytokine released from T cells, NK cells, monocytes, and epithelial cells. Recently, IL-32 has been shown to be related with autoimmune diseases, but the role of IL-32 in *H pylori* infection is still unclear. In the literature, there are a limited number of studies about IL-32 in malignancies. However, earlier studies have shown an increase in IL-32 expression in gastric, hepatocellular, and lung carcinomas.^38–40^ Dissimilar to previous studies, our study showed no significant differences in the mucosal genomic expression of IL-32 with respect to the presence of malignancy or inflammation (Figure 8). IL-32 may not play a role in *H pylori* infection and related GAC risk. Further prospective studies should be conducted to determine the clinicopathological significance of IL-32 as a predictive marker of *H pylori*–related GACs.

D’elios et al researched *H pylori*–specific cytokine pattern and demonstrated IFN-γ, TNF-α, and IL-12 but no IL-4 mRNA expression, pointing out the Th1-type response. They concluded that Th1-derived cytokines induce macrophages to release proinflammatory cytokines causing the tissue damage.^41^ Similarly, in our study, both CAG and GAC groups showed Th1-type response predominantly with also both groups showing a Th17 profile.

The main limitation of our study is that *H pylori*–negative patients and those with low-stage GAC were not included; therefore, we could not obtain data about the low stages of gastric cancer and the difference between *H pylori*–negative and –positive diseases. Owing to cross-sectional nature of this study, we cannot address predictive or causative markers.

How *H pylori* infection is associated with carcinoma is still not clear. Our findings suggest that the immune response of gastric mucosa to *H pylori* varies among patients. Increased genomic expression of cytokines in chronic gastritis is expected because of increased immune cell infiltration. In contrast, lower levels of expression in GAC developing from an atrophic mucosa reflect gastric glandular reduction and lower levels of immune cell infiltration. In this study, genomic expression level**s** of some proinflammatory cytokines such as IFN-γ decreased contrary to increased levels of IL-6.
